# Two-Person Neuroscience and Naturalistic Social Communication: The Role of Language and Linguistic Variables in Brain-Coupling Research

**DOI:** 10.3389/fpsyt.2014.00124

**Published:** 2014-09-05

**Authors:** Adolfo M. García, Agustín Ibáñez

**Affiliations:** ^1^Laboratory of Experimental Psychology and Neuroscience (LPEN), Institute of Cognitive Neurology (INECO), Favaloro University, Buenos Aires, Argentina; ^2^National Scientific and Technical Research Council (CONICET), Buenos Aires, Argentina; ^3^School of Languages, National University of Córdoba, Córdoba, Argentina; ^4^UDP-INECO Foundation Core on Neuroscience (UIFCoN), Diego Portales University, Santiago, Chile; ^5^Universidad Autónoma del Caribe, Barranquilla, Colombia; ^6^Australian Research Council Centre of Excellence in Cognition and its Disorders, Sydney, NSW, Australia

**Keywords:** social cognition, two-person neuroscience, interpersonal communication, language, dialog

## Abstract

Social cognitive neuroscience (SCN) seeks to understand the brain mechanisms through which we comprehend others’ emotions and intentions in order to react accordingly. For decades, SCN has explored relevant domains by exposing individual participants to predesigned stimuli and asking them to judge their social (e.g., emotional) content. Subjects are thus reduced to detached observers of situations that they play no active role in. However, the core of our social experience is construed through real-time interactions requiring the active negotiation of information with other people. To gain more relevant insights into the workings of the social brain, the incipient field of two-person neuroscience (2PN) advocates the study of brain-to-brain coupling through multi-participant experiments. In this paper, we argue that the study of online language-based communication constitutes a cornerstone of 2PN. First, we review preliminary evidence illustrating how verbal interaction may shed light on the social brain. Second, we advance methodological recommendations to design experiments within language-based 2PN. Finally, we formulate outstanding questions for future research.

## Introduction

Human brains are social entities. These organs learn, (re)construct, and use information in interactive multi-participant contexts. Our daily life depends on the ability to comprehend others’ emotions and intentions in order to react accordingly. These skills are the object of study of social cognitive neuroscience [SCN, Ref. (1–3)]. Here, we discuss the limitations of mainstream SCN and highlight the benefits of studying language-based social interactions to develop a naturalistic approach to the field. Moreover, we advance methodological recommendations and formulate outstanding research questions.

Social cognitive neuroscience has focused on socially relevant cognitive domains, such as emotional processing, empathy, and theory of mind. A wealth of empirical evidence has shown that these functions are partially dissociated at a behavioral level (1, 4) and subserved by specific brain networks, such as the amygdala network, the mentalizing network, the empathy network, and the action–perception network (5). Other networks, such as the salience network (6) or the social context network (7), have been implicated in general domains affecting multiple social-cognition areas. Electroencephalography evidence has shown that alpha and theta oscillations are sensitive to social risk and play a regulatory role in social decision-making (8). SCN findings such as these have been used to postulate models of the social brain and to advance clinical and therapeutic proposals (5, 9). However, the very validity of standard research within SCN has recently come under fire.

Most social-cognition experiments employ isolation paradigms (10), that is, behavioral, neurophysiological, or neuroimaging set-ups in which an individual participant is shown static (e.g., pictures, words) or dynamic (e.g., videos) stimuli and asked to categorize/judge their contents or infer the mental/emotional states of their protagonists. Subjects are thus reduced to detached observers of situations that they play no active role in. Accordingly, most SCN models are tacitly or otherwise committed to a “spectatorial view of social knowing,” which frames interpersonal contact as a unidirectional, non-interactive phenomenon (11).

Admittedly, there are everyday scenarios in which we activate our social-cognition systems while assuming a spectator’s role (e.g., while watching a movie, reading a comic book, or looking at two people arguing on the street). However, these are peripheral to the core of our social experience, which is mostly construed through interactions requiring active negotiation of unpredictable, implicit, multimodal information. If the neural basis of social knowledge is to be explored more directly and ecologically, mainstream methods must be complemented with an interactional approach to SCN. This epistemological recast has been dubbed as “second-person” (11) or “two-person” (12) neuroscience (2PN).

## 2PN: Principles and Evidence

Two-person neuroscience advocates the study of brain-to-brain coupling (13) to erect a neurocognitive science of social interaction (14). This framework assumes that the neurobiological mechanisms subserving detached assessment of social phenomena are different from those activated in emotionally engaged interactors. Schilbach et al. (11) argue that social interactions involve complex reciprocities between agents who (re)act to relevant information conveyed by their counterparts and are aware of the resources that they hold collectively. The authors further highlight three key aspects of 2PN: (i) the interactors may dynamically assume different roles (e.g., initiator and responder); (ii) interactions give rise to new shared intentions and motivations; and (iii) social relations are constrained by the interactors’ histories (i.e., their developmental neuropsychological trajectories). Moreover, an interaction’s contextual background (from physical situation to social embeddedness) features continuous changes, which are not signaled by explicit rules.

Although 2PN poses major methodological challenges, which are not part of spectatorial SCN, several studies have offered valid, reliable data through interactive paradigms. In Schilbach et al.’s (15) study, a virtual character exhibited facial expressions on a computer screen. Some were socially relevant and some were arbitrary. At certain times, the character looked directly at the participant; at others, it directed its gaze sideways to another agent. The participants effectively discriminated between socially relevant and arbitrary expressions. Interestingly, brain imaging data showed that self-directed expressions differentially increased activity in the medial prefrontal cortex and superficial amygdala, two regions implicated in emotional and evaluative processing.

Particularly interesting are studies involving hyperscanning (16), a technique which allows to simultaneously acquire neural data from two participants through dual electroencephalography or functional magnetic resonance imaging (fMRI). Hyperscanning data have shown that participants’ brains synchronize their activity in the same regions during interactive tasks. Using a finger-movement task, Yun et al. (17) showed that when subjects moved their fingertips in a coordinate fashion, a frontoparietal network was synchronically activated in both their brains. The authors proposed that fingertip movement synchronization could index implicit interpersonal interaction. Furthermore, they surmised that the inferior frontal cortex, together with the anterior cingulate cortex and the parahippocampal and postcentral gyri, might constitute the neural basis of inter-brain synchronization.

The synchronized finger-tapping task was also used by Konvalinka et al. (18), who examined leader–follower interaction through dual electroencephalographic recordings. Participants had to adjust their finger-tapping patterns either to each other or to a computer metronome. Relative to the latter, the interactive condition revealed a stronger suppression of alpha and low-beta oscillations over motor and frontal areas. Interestingly, frontal alpha suppression was stronger in participants assuming the role of leaders. The authors suggest that leaders invest more cognitive resources in planning and control, and that the spontaneous establishment of leader–follower relationships can be predicted from brain activity both before and during interaction.

In a more complex hyperscanning experiment, Saito et al. (19) combined eye-tracking, video cameras, and fMRI to obtain brain data from two participants who interacted with their partner’s real-time avatar on a computer screen. Participants were asked to either look at (or away from) colored targets or to follow (or detract from following) the interactor’s gaze toward a given target. A crucial finding was that, relative to non-paired subjects, paired subjects evinced higher neural correlations in the right inferior frontal gyrus. This region, the authors propose, may support shared intentionality and joint attention.

These preliminary findings show that direct interpersonal engagement modulates the neurocognitive patterns associated with social cognition. Active participation in a multi-agent scenario calls for specific neural mechanisms and favors brain coupling. Contrary to standard SCN, 2PN directly addresses the underpinnings of interpersonal engagement. In this sense, of critical importance to 2PN is the domain of verbal communication, as discussed below.

## Language-Based Communication as a Cornerstone of 2PN

Language-based communication has been acknowledged as a cornerstone of 2PN (11, 20). Systemic-functional linguists have defined language as a social semiotic (21), one of whose main functions is to establish, maintain, and modify social relations (22). Proponents of the situated cognition approach have shown that linguistic meanings are highly negotiable (23). Cognitive neuroscientists have argued that an utterance’s meaning depends on ongoing body actions and social interaction (24, 25). Developmental psychologists highlight verbal communication as a manifestation of a drive to share intentions and knowledge (26). As it is inherently dialogical, language constitutes a main focus of interest to 2PN.

Like SCN, psycholinguistics and neurolinguistics have largely neglected the interactive nature of their domain of inquiry. Undoubtedly, both disciplines have produced invaluable evidence for theoretical and translational aims. However, the most fundamental features of language remain underresearched within cognitive neuroscience, let alone SCN.

Bodily action systems, which mediate varied social processes, are functionally coupled with neural language networks. Ongoing actions are interwoven with verbal production and comprehension, as these processes are forms of action and action perception (27). Joint action serves as a blueprint for dialog coordination and the experience of shared reality (28). A bidirectional relationship is frequently observed between action and language, including a crosstalk effect (29). The level of congruency between current actions and active semantic representations affects both motor and linguistic processes at brain level (30, 31). Moreover, such a bidirectional coupling seems to be early impaired in motor diseases, such as Parkinson’s or Huntington’s disease (32–35). All this evidence indicates that linguistic processes are closely related to an individual’s ongoing activity. This active loop may play a critical role in coordinating brain activity between interlocutors.

As regards social cognition, SCN has demonstrated the impact of language on emotion processing [for a review, see Ref. (36)]. Barrett (37) hypothesized that emotion words can guide children’s acquisition of emotion categories and influence emotion assessment throughout life. In emotion recognition tasks, accuracy is significantly reduced when participants are not given a closed set of labels to choose from (38). This suggests that linguistic information constrains the appraisal of others’ emotional states.

Roberson and Davidoff (39) reported that perceptual judgment of faces is disrupted by concurrent word processing. Similarly, when subjects repeat an emotion word (e.g., *anger*) 30 times before deciding whether two faces show the same emotion, their speed and accuracy is significantly reduced (40). Such effects may indicate that competing lexical activations interfere with access to the words needed to produce an adequate judgment. Furthermore, a comparison of brain potentials evoked by perception of emotion-laden faces showed that P1 and N170 components were differentially sensitive to expressions of anger and fear only when participants had to explicitly categorize them under a verbal label (41). These components may index language effects on emotion discrimination.

In sum, language seems to mediate the processing of both ongoing actions and social-cognition processes. However, while these findings are relevant for language-based 2PN, they stem from isolation paradigms. More direct evidence comes from studies exploring the role of language in interactional settings.

Language shapes and reflects social roles while engaging social-cognition domains. For example, communicative roles are at constant interplay in verbal exchanges. Within seconds, unfolding discourse choices alternately construe interlocutors as information givers or information seekers among other roles (42). Mothers guide their children’s attention by using predictable prosodic patterns (43). Adults make phonological adjustments during dialog to approach their interlocutors’ accents (44). Bilingual speakers must assess their interlocutors’ sociocultural background to establish which language to use and whether code-switching is permissible (45).

Interpersonal communication can rely on several resources, such as gestures, facial expressions, or language. In the latter case, it is known as dialog. Experimental research on dialog demonstrates that social interaction is sensitive to cross-subject effects. Branigan et al. (46) asked pairs of participants to alternately describe pictures to each other. One speaker was trained to characterize the pictures following scripts that systematically varied in syntactic structure. Crucially, it was found that this speaker’s syntactic choices influenced those of his counterpart, through a form of syntactic priming. Cooperative conversation was also shown to favor postural coordination between interlocutors. Shockley et al. (47) reported that shared postural activity increased at faster speaking rates and by production of the same (or similarly stressed) words. Notably, this effect emerged only when participants’ data were compared to those of a partner who was present during the task.

Using fMRI, Stephens et al. (48) obtained brain data from both speakers and listeners during naturalistic conversation. Brain activity between interlocutors was spatiotemporally coupled during successful communication, but such a coupling vanished when communication failed (e.g., when listening to an unintelligible foreign language). Also, the more extensive the neural coupling was, the more successful the communication proved. Finally, anticipatory activity was detected in the listener’s brain, particularly in the striatum and the medial and dorsolateral prefrontal cortices – regions associated with anticipatory skills.

In another hyperscanning study, Jiang et al. (49) measured brain activity in participant pairs performing four communicative tasks: face-to-face dialog, back-to-back dialog, face-to-face monolog, and back-to-back monolog. Whereas face-to-face dialog significantly increased inter-brain synchronization in the inferior frontal cortex, no synchronization was observed in the remaining conditions. The authors concluded that face-to-face communication has distinctive neural features associated with the integration of multimodal sensory information and turn-taking behavior.

Language evinces sensitivity to social contact from early age, as shown by Kuhl et al. (50). In their first experiment, 9-month-old American infants listened to native Mandarin speakers in multiple sessions, while a control group was exposed to English. In a second experiment, the infants were exposed to the same speakers and materials, but this time through videos and audio recordings. The main finding was that the infants learned Mandarin phonemes in the first condition, but not in the second one. Thus, the authors proposed that phonetic learning is enhanced by social interaction.

Taken together, these studies show that language-based communication incarnates the tenets of 2PN. A speaker’s linguistic choices are influenced by those of his/her interlocutor and, when these coincide, postural coordination increases. Also, the synchrony between two interlocutors’ brains is sensitive to communicative success and engagement level. Finally, social contact seems to enhance language acquisition.

Two-person neuroscience offers sound alternatives to overcome some of the limitations of standard SCN. In this sense, social brain-coupling research should not be blind to the fact that most social interactions are mediated by language. In fact, language often guides and regulates interaction dynamics, as dialog supports the establishment of goals, subgoals, and patterns of agreement during joint activities (51). All in all, 2PN should rely heavily on experiments exploring or including linguistic exchanges, as verbal interaction may offer critical insights into the workings of the social brain.

## Methodological Recommendations

The design of interactive, language-based experiments calls for distinct methodological precautions. First, key linguistic variables should be controlled for. In addition to typical psycholinguistic measures, such as word frequency, 2PN experiments must pay attention to broad aspects of verbal communication, including field, tenor, and mode (22). Field refers to the relations between the subject matter and the social processes that it can give rise to. It is critical to decide, which issues will be proposed for discussion between participants, as conversation topics differ in their level of social commitment (high in debating capital punishment and low in discussing weather), the semantic fields they activate, and the lexicogrammatical choices they tend to favor. Tenor concerns the social roles and relations of the interactors. In this sense, researchers must note that different interpersonal configurations (e.g., expert-to-expert, expert-to-layman, layman-to-layman) modify the participants’ linguistic choices (e.g., clause type) and communicative moves (e.g., giving information, asking for clarification). Mode involves variables such as medium (e.g., spoken or written) and the channel (e.g., face-to-face, telephonic, computer chat-based) of the exchange. These aspects affect the speed of delivery, the degree of implicitness admissible, and, more importantly, the amount and type of cross-participant feedback and the level of emotional engagement. In sum, language-based 2PN research should carefully specify the field, tenor, and mode governing the verbal exchanges under study.

Second, special note should be taken of the language(s) used in each study. Just like the same underlying linguistic deficit may have different surface manifestations across languages (52), so could the same socio-interactive process have varied language-specific realizations. For instance, the contrast between statements and questions is marked through syntax in English but through suprasegmental phonology in Spanish (53). Also, several Indo-European languages possess different pronouns for the second-person singular (e.g., *tu/vous* in French, *du/Sie* in German, *tu/lei* in Italian, and *tú/usted* in Spanish, and *vos/tú/usted* in Ríoplatense Spanish). The choice of one or the other depends on the perceived social distance between the speaker and addressee or even on contextual constrains (54). Some languages even code the perception of an interlocutor’s attentional engagement. For example, the Colombian language Andoke features grammatical auxiliaries indicating whether the speaker considers that the addressee is attending to the event represented by an utterance (55). Furthermore, in several languages, communicative roles are dynamically and automatically assigned by specific word classes. In English, the occurrence of a *wh-* word at the beginning of an interrogative sentence ascribes speaker and hearer the roles of “information seeker” and “information giver,” respectively. Similarly, the use of a question tag after a statement renders the speaker a “confirmation seeker” (42). In Japanese, different grammatical constructions reflect epistemic asymmetries, such as the distinction between information stemming from oneself (e.g., feeling sad) and information derived from observation (e.g., outward signals of sadness). The structural idiosyncrasies of the language(s) used in an experiment should not be overlooked when designing language-based 2PN studies or when interpreting their results.

Third, to maximize the usefulness of linguistic and social-cognition data, tasks and stimuli should limit the scope of possible interactive behaviors. Otherwise, irrelevant conducts might render the data uninformative for a specific research question. Consider the study by Coventry et al. (56), who explored the relationship between extrapersonal space and language by focusing on demonstrative pronouns – i.e., words indicating how proximal or distal a referent is perceived to be. By restricting participants’ responses to either *this* or *that*, the authors demonstrated that the use of the former to refer to an object was more frequent when the object was placed by the participant rather than the experimenter. This study demonstrates that the linguistic construal of space is sensitive to the action of other individuals and, more generally, illustrates the empirical benefits of restricting the range of verbal responses in language-based 2PN.

Fourth, a more ecological way of controlling (aspects of) the participants’ production is to use a covert “confederate,” that is, an interactor who has been previously trained to elicit or induce specific responses from the actual participant. This strategy allows researchers to control important variables while creating an interactive setting, which the real participant will perceive as spontaneous. A good illustration of this strategy is Branigan et al.’s (46) study on cross-subject syntactic priming (see Language-Based Communication as a Cornerstone of 2PN). Alternatively, the use of a computer-controlled character (or even an avatar) could enhance experimental control while prompting genuinely interactive behaviors (15, 19). In studies using human interactors, the level of acquaintance should be controlled between pairs (49). These recommendations should be considered in approaching the outstanding questions for future research listed in Table 1.

**Table 1:** **Outstanding questions for future research**.

Empirical/theoretical basis	Observation	Question
Brain coupling between non-related interlocutors increased during successful, as opposed to unintelligible exchanges (48) and during face-to-face conversation, as opposed to monolog and back-to-back dialog (49)	As opposed to unrelated individuals, parents and children, husbands and wives, brothers, friends, and even co-workers share developmental trajectories, which may shape their interaction patterns	Do the neural mechanisms underlying communicative interaction and interpersonal synchronization vary as a function of kinship?
Evidence from isolation paradigms shows that utterances conveying literal and figurative meanings engage different neurocognitive mechanisms (57)	The neural mechanisms subserving the observation of social phenomena differ from those active in emotionally engaged interactors (11)	Do the neural responses to literal and figurative language differ when the utterances are displayed on a computer or produced by a person in a real context?
Evidence from isolation paradigms shows that linguistic processing modulates emotion recognition (36)	The neural mechanisms subserving the observation of social phenomena differ from those active in emotionally engaged interactors (11)	Is emotion recognition differently modulated by computer-displayed linguistic stimuli and face-to-face, human-made utterances?
Face-to-face conversation, as opposed to monolog and back-to-back dialog, increased neural synchronization between participants (49)	Medium (e.g., spoken or written) and channel (e.g., face-to-face, telephonic, computer chat-based) shape social relations during verbal exchanges (22)	Do different forms of real-time verbal interaction (face-to-face dialog, phone conversation, computer-based chat) modulate the mechanisms supporting inter-participant brain coupling?
The tenor of an exchange varies as a function of the social roles and relations of the interactors (22)	Interlocutor pairs may present different levels of demographic symmetry/asymmetry (e.g., in terms of gender and age)	Do the level and the substrates of neural coupling between interlocutors vary as a function of their demographic symmetry (same-sex pair, opposite-sex pair, child–child, adult–adult, child–adult)?
Specific sentence types automatically assign communicative roles to the interlocutors (e.g., a question renders the speaker an information seeker and the addressee and information giver) (42)	In dialog, communicative roles can fluctuate (e.g., in a casual conversation, two friends alternate roles as information seekers and information givers) or remain constant (e.g., in an oral exam, the teacher monopolizes the role of information seeker and the student is framed as an information giver)	Do the level and the substrates of neural coupling between interlocutors vary as a function of the fixedness of their communicative roles?
Evidence from isolation paradigms indicates that affective processing in bilinguals is modulated by the language they use (58)	Bilinguals may communicate with other bilingual speakers or with monolingual users of either their native or non-native language	Do the level and the substrates of neural coupling between interlocutors change when one or both of them communicate in a non-native language?

## Conclusion

Social cognitive neuroscience has furthered our understanding of multiple socially relevant aspects of neurocognition. However, it has failed to directly address the core of social relations, namely, the deployment of interactive processes between emotionally engaged participants in contextually dynamic environments. 2PN seeks to bridge such a gap by exploring inter-brain communication in multi-participant experimental settings. We propose that the study of language, in general, and online dialog, in particular, constitutes a cornerstone of this incipient field. Current knowledge about the social brain could be dramatically broadened by future investigations on inter-individual verbal communication.

## Conflict of Interest Statement

The authors declare that the research was conducted in the absence of any commercial or financial relationships that could be construed as a potential conflict of interest.
